# Septic shock due to *Clostridium botulinum*: a case report

**DOI:** 10.1186/s13256-023-04044-3

**Published:** 2023-09-07

**Authors:** P. M. L. Zomer, M. J. A. Kamps

**Affiliations:** Department of Intensive Care Medicine, Catharina Hospital Eindhoven, Michelangelolaan 2, 5623 EJ Eindhoven, The Netherlands

**Keywords:** Case report, Septic shock, Infectious disease, *Clostridium botulinum*, Botulism, Gram-positive bacteria

## Abstract

**Background:**

*Clostridium botulinum* is an anaerobic, Gram-positive, rod-shaped bacterium that forms spores and the neurotoxin botulinum. It is best known for its toxin-induced flaccid paralytic disease, which is deadly without correct treatment. In this report, we show a completely different clinical course with fatal outcome.

**Case presentation:**

A 37-year-old African man born in Sierra Leone was admitted. After admission, his condition rapidly worsened due to severe septic shock and progressive multiorgan failure. No neurological signs were seen. Despite treatment with antibiotics, fluid resuscitation, and norepinephrine, the multiorgan failure deteriorated further and he died as a result. Blood and synovial fluid cultures showed *Clostridium botulinum*. No botulinum toxins were found.

**Conclusion:**

This is a rare case of fatal septic shock due to *Clostridium botulinum*-induced septic arthritis without any sign of the classic clinical syndrome of botulism.

## Introduction

*Clostridium botulinum* is known to produce the botulinum toxin, which is a highly toxic substance and lethal to humans even at low doses [1]. *C. botulinum* is an anaerobic Gram-positive spore-forming rod and is the most common cause of botulism. Botulism includes the clinical syndrome of symmetrical cranial nerve palsies followed by descending, symmetric flaccid paralysis of voluntary muscles, which may progress to respiratory compromise and without correct treatment to death [2]. The manifestations of botulism are due to the botulinum neurotoxin produced by *C. botulinum*, which blocks presynaptic acetylcholine release. This inhibits muscle contraction and causes flaccid paralysis [3, 4]. We present, to our knowledge, the first case of fatal septic shock caused by a *C. botulinum* arthritis in an immunocompetent patient without any of the classic neurologic signs.

## Case presentation

**Fig. 1:**
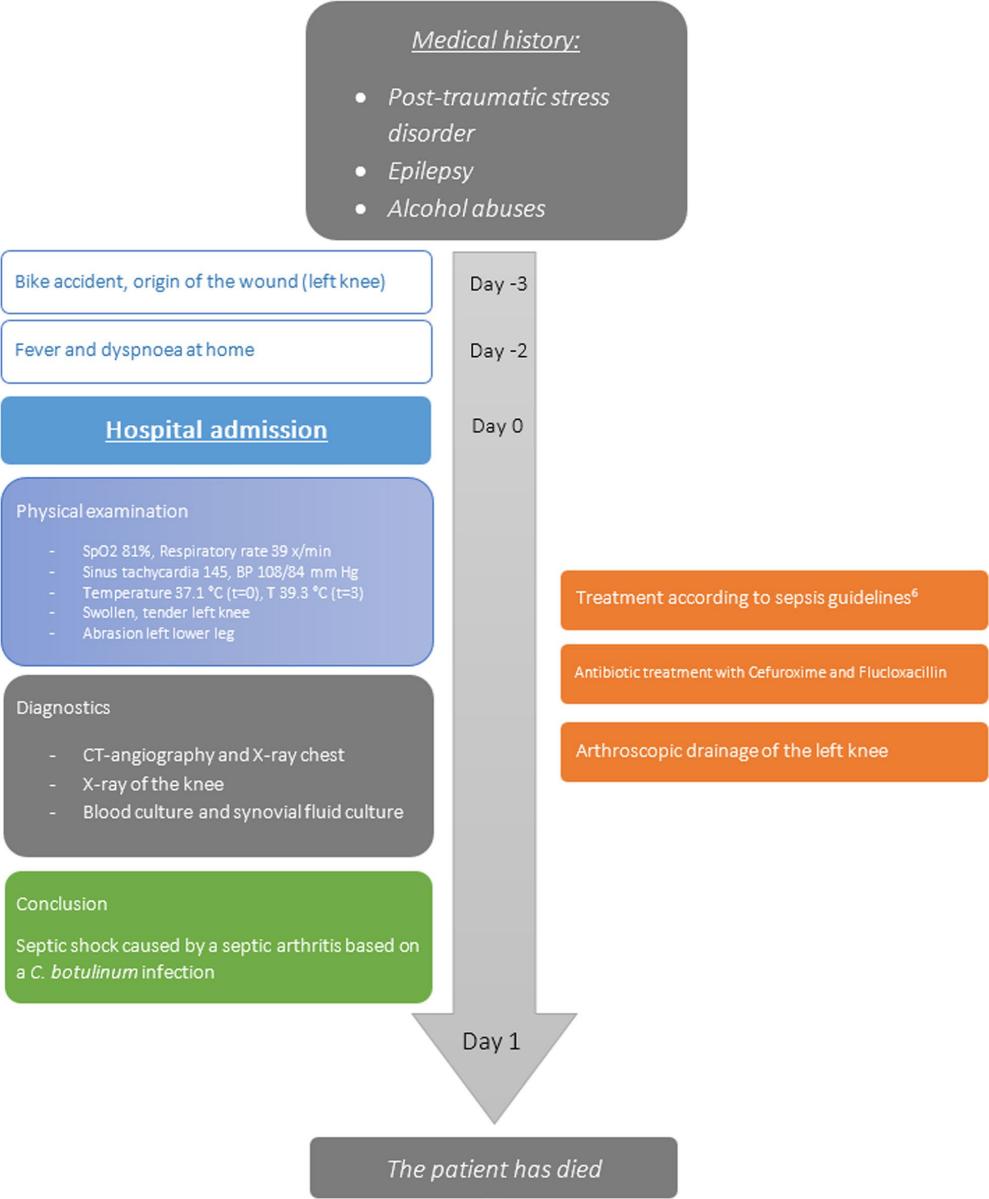
Timeline graph of the main events in our patient’s disease course

A 37-year-old man presented to our emergency department (ED) with recent history of fever (Fig. 1). He was born in Sierra Leone and of African ethnicity, and had lived in the Netherlands since 2005. History revealed that the patient had fallen on his left knee 3 days ago after he collided with a car while riding a bicycle. He had a superficial skin defect just below his left knee from scraping the pavement. There was no penetration of the knee joint. Since then he had a swollen left knee and a sore right hip. In the days prior to presentation to the ED, the pain increased and he developed progressive dyspnea. His prior medical history included posttraumatic stress disorder, alcohol abuse, and epilepsy, of importance he reported no prior joint swelling or pain. Furthermore, he had no recent travel history and he did not smoke or use drugs.

**Fig. 2:**
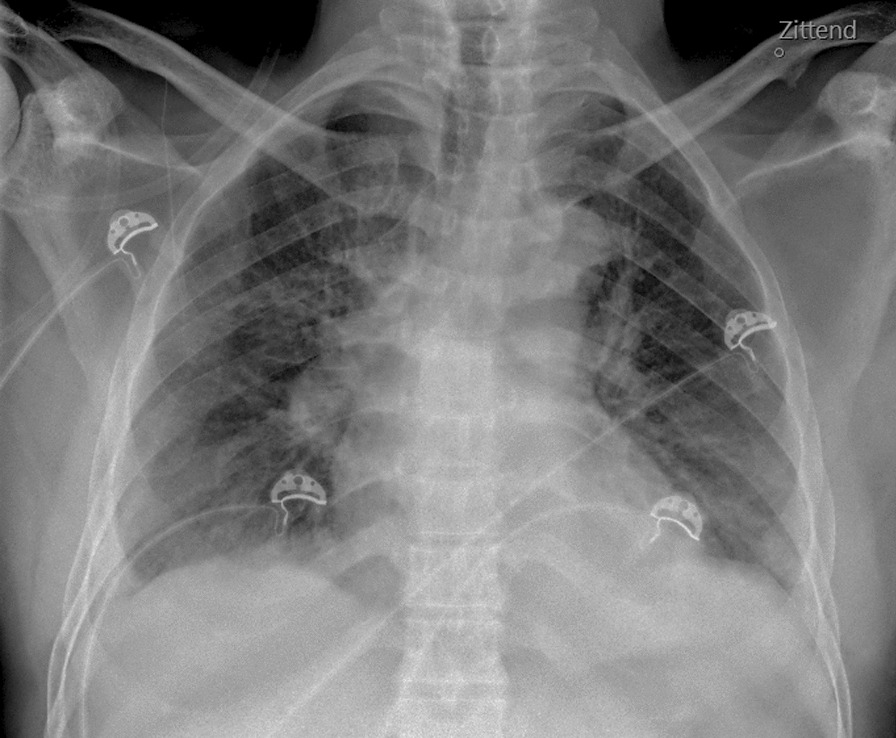
Chest X-ray with pleural effusion and bilateral peripheral consolidations

During examination there was a free airway with a respiratory rate of 39/minute with oxygen saturation of 81% on room air. Chest X-ray showed bilateral pleural effusion with bilateral peripheral consolidations (Fig. 2). His blood pressure was 109/85 mmHg with a pulse of 145 bpm. Transthoracic ultrasound showed normal left and right ventricular function without any valve abnormalities. Despite a mildly decreased Glasgow Coma Scale of 13, no other neurologic symptoms were observed, in particular no sign of meningism or other neurological abnormalities such as symmetric paralysis of limbs, head, and facial musculature. Glucose levels were normal. Initially, the temperature was 37.1 °C but a few hours after admission temperature rose to 39.3 °C. The most remarkable finding during physical examination was an extremely painful, swollen left knee with an abrasion on the left lower leg.

**Fig. 3:**
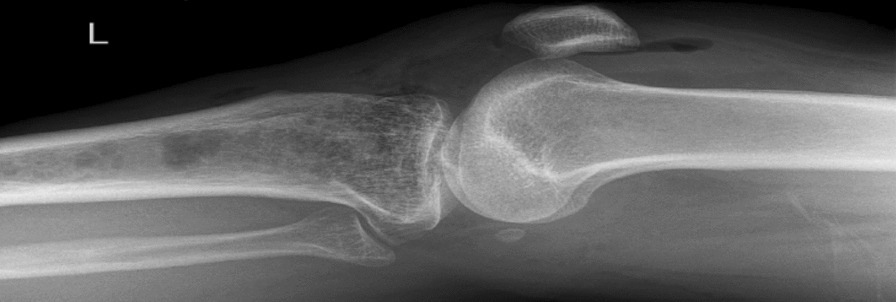
X-ray of the left knee shows osteolytic bone lesions of the tibia and gas in the suprapatellar pouch

Laboratory findings on admission revealed a C-reactive protein of 460 mg/L, leukocyte count of 11.8 × 10^9^/L with severe acute renal insufficiency (creatinine 245 µmol/L), arterial blood gas with metabolic acidosis based on a lactate of 6.6 mmol/L and a bicarbonate level of 19.0 mmol/L. X-ray of the left knee showed osteolytic bone lesions of the tibia and gas in the suprapatellar pouch (Fig. 3). No blood samples were taken on admission to measure ethanol levels. Based on these findings, we concluded that he developed severe shock with organ dysfunction, but without meeting the definition of septic shock at that moment according to the Sepsis-3 guidelines [5]. This was probably caused by bacterial arthritis or as a differential diagnosis as a result of pneumonia based on the dyspnea and chest X-ray. Pulmonary embolism and tuberculosis were ruled out by computed tomography (CT) angiography of the chest.

The patient was treated according to the Surviving Sepsis guideline [6]. Blood cultures were taken, and cefuroxime was administered within an hour after presentation at the emergency department, as well as administration of a tetanus vaccine. Based on physical examination and X-ray of the left knee, a bacterial arthritis was deemed most likely. Therefore, an arthroscopy was performed to confirm the diagnosis and perform a debridement. During the procedure, high quantities of pus were removed. Antibiotic treatment with flucloxacillin was started, according to the local empirical treatment of septic arthritis. In addition, thiamine was given due to the history of alcohol abuse. Postoperatively he developed severe septic shock with rapidly progressive multiorgan failure including acute respiratory distress syndrome (ARDS) with severe hypoxemia [7]. We treated the hypoxemia with mechanical ventilation and the shock by resuscitation with fluids and high doses of norepinephrine. Despite all efforts, the patient died. Blood cultures (Biomerieux Bact/ALERT^®^), on admission and day 1, and synovial fluid cultures (local routine culture methods) grew *C. botulinum* after 1 day of incubation without any other pathogens. Sequencing (16S) of aspirated synovial fluid of the left knee showed *C. botulinum*. Autopsy was performed, no other cardio, pulmonary or internal organ abnormalities were found. A small superficial abrasion just below the tense swollen left knee showed yellow pus. Cross section of the tibia behind the abrasion showed a lytic lesion.

## Discussion

We report a rare case of fatal septic shock due to *C. botulinum*-induced septic arthritis. *C. botulinum* is known for its neurotoxin production, which causes botulism. There are various types of botulism. Wound botulism is caused by *C. botulinum* colonization of a wound and *in situ* toxin production. Infant botulism is caused by intestinal colonization and toxin production and is quite rare. Adult intestinal toxemia botulism is an even rarer form of intestinal colonization and toxin production in adults. Inhalational botulism results from aerosolization of botulinum toxin, and iatrogenic botulism can result from injection of toxin. All forms of botulism produce the same distinct clinical syndrome of symmetrical cranial nerve palsies followed by descending, symmetric flaccid paralysis of voluntary muscles, which may progress to respiratory compromise and death [2, 8]. It has generally been thought that extremity weakness precedes respiratory distress or failure. However, some cases have been described whereby respiratory involvement occurred early in the illness without preceding extremity weakness. Besides, cases are reported with presentation of respiratory features without cranial nerve palsies. Other patients developed cranial nerve palsies on hospital day 2 or 3 [9].

Botulism treatment involves supportive care, intubation, and mechanical ventilation when necessary. If patients present with the aforementioned neurological signs and botulism is suspected, botulinum antitoxin must be administered as quickly as possible as it is the only effective treatment for botulism. Circulating neurotoxins can be bound to botulinum antitoxin, which prevents their binding to the neuromuscular junction. It cannot reverse paralysis, thus the administration needs to occur early in the disease course. In addition, debridement is generally performed in wound botulism. Clinical guidelines mention that treatment should address each patient’s clinical situation [8].

In this case, the patient presented with arthritis of the left knee and respiratory insufficiency without any neurological signs. Initially, the suspicion for *Staphylococcus aureus* bacteremia caused by wound infection of the left knee was high, as the most probable causative bacterium for arthritis is *S. aureus*, followed by other Gram-positive cocci [10]. Therefore, according to our local protocol, continuous flucloxacillin was started. Because of the fulminant course, we considered whether the patient could be immunocompromised. He tested negative for human immunodeficiency virus (HIV) and had normal immunoglobulin levels (IgG 9.4 g/L and IgM 0.99 g/L). One day after admission, both blood and synovial fluid cultures were positive for *C. botulinum*. Furthermore, sequencing (16S) of the synovial fluid confirmed *C. botulinum*. Before cultures revealed additional information about the source of infection, the patient deteriorated fast and died. Afterwards, testing for botulinum neurotoxins was done by the mouse bio-assay. No botulinum neurotoxins were detected.

*C. botulinum* colonization in wounds may ultimately lead to botulism through the formation of the *C. botulinum* neurotoxin. However, in this case there was no presentation with classic neurologic signs seen in botulism and no *C. botulinum* neurotoxins were detected, and a typical bacterial septic shock developed. If the clinical manifestation of botulism had stood more out, botulinum antitoxin could have been administered. Taken together, this clinical presentation did not appear to be fully associated with the clinical syndrome of wound botulism, and therefore we did not give the botulinum antitoxin [2]. On the other hand, this clinical presentation did meet the criteria for septic shock caused by *C. botulinum* infection. Sepsis is defined as life-threatening organ dysfunction caused by a dysregulated host response to infection, whereby patients with septic shock can be clinically identified by a vasopressor requirement to maintain a mean arterial pressure (MAP) of 65 mmHg or greater and serum lactate level greater than 2 mmol/L in the absence of hypovolemia [5]. This patient met the qSOFA criteria [5], had organ dysfunction with respiratory insufficiency and acute kidney injury, had inadequate tissue perfusion resulting in lactate acidosis, and eventually needed vasoactive drugs to maintain a MAP above 65 mmHg. Antibiogram (susceptibility testing performed with MIC gradient strips), available after the patient died, showed that the isolated *C. botulinum* was susceptible to penicillin and cephalosporins. This indicates that he was properly treated at admission with cefuroxime and later with flucloxacillin, but this could not prevent the detrimental course of his disease. Earlier presentation and initiation of adequate therapy could have influenced our patient’s disease course.

## Conclusion

This case of rapidly progressive fatal septic shock occurred in an immunocompetent patient with a Gram-positive rod-shaped bacterium, *C. botulinum*. His skin lesion was merely a superficial abrasion. To our surprise, cultures showed *C. botulinum*. This bacteria is mostly known for its induction of generalized paralysis and respiratory muscle failure caused by the neurotoxin botulinum. If these clinical features are present, rapid treatment with antitoxin is warranted. In contrast, these symptoms can also be absent, probably because this toxin is not produced in this particular case. It is important to note that infection with this bacteria can be fatal without its infamous botulinum neurotoxin production but by inducing fulminant and fatal sepsis.

## Data Availability

Data sharing is not applicable to this article as no datasets were generated or analyzed during the current study.
